# Identification of sequence mutations in *Phytophthora cactorum* genome associated with mefenoxam resistance and development of a molecular assay for the mutant detection in strawberry (*F.* × *ananassa*)

**DOI:** 10.1038/s41598-023-34271-z

**Published:** 2023-05-06

**Authors:** Marcus V. Marin, Juliana S. Baggio, Youngjae Oh, Hyeondae Han, Saket Chandra, Nan-Yi Wang, Seonghee Lee, Natalia A. Peres

**Affiliations:** 1 Department of Plant Pathology, University of Florida, Gainesville, FL 32611 U.S.A.; 2 Gulf Coast Research and Education Center, University of Florida, Wimauma, FL 33598 U.S.A.; 3 Department of Horticultural Science, Chungbuk National University, Cheongju, South Korea; 4 School of Biotechnology, National Institute of Technology Calicut, Kozhikode, India; 5 Horticultural Science Department, University of Florida, Gainesville, FL 32611 U.S.A.

**Keywords:** Plant sciences, Microbiology

## Abstract

Phytophthora crown rot (PhCR) caused by *Phytophthora cactorum* is one of the most damaging diseases of strawberry worldwide. Mefenoxam is one of the major fungicides currently used to manage PhCR. However, the emergence and spread of resistant isolates have made controlling the pathogen in the field problematic. In the present study, using whole genome sequencing analysis, mutations associated with mefenoxam-resistant isolates were identified in six different genomic regions of *P. cactorum*. The 95.54% reads from a sensitive isolate pool and 95.65% from a resistant isolate pool were mapped to the reference genome of *P. cactorum* P414. Four point mutations were in coding regions while the other two were in noncoding regions. The genes harboring mutations were functionally unknown. All mutations present in resistant isolates were confirmed by sanger sequencing of PCR products. For the rapid diagnostic assay, SNP-based high-resolution melting (HRM) markers were developed to differentiate mefenoxam-resistant *P. cactorum* from sensitive isolates. The HRM markers R3-1F/R3-1R and R2-1F/R2-1R were suitable to differentiate both sensitive and resistant profiles using clean and crude DNA extraction. None of the mutations associated with mefenoxam resistance found in this study were in the RNA polymerase subunit genes, the hypothesized target of this compound in oomycetes. Our findings may contribute to a better understanding of the mechanisms of resistance of mefenoxam in oomycetes since serves as a foundation to validate the candidate genes as well as contribute to the monitoring of *P. cactorum* populations for the sustainable use of this product.

## Introduction

Florida is the largest strawberry winter producer, being responsible for more than 5% of the U.S. production^1^. In 2020, Florida produced approximately 89,000 tons of strawberry over its 4000 harvested hectares raising 240 million dollars^2^. Unfortunately, crop losses caused by plant pathogens represent one of the major threats in strawberry production and agriculture in general and have become even more challenging since organisms are prone to selection for resistance to single-site fungicides commonly used for their control.

Phytophthora crown rot (PhCR), caused mainly by *Phytophthora cactorum*, is an important disease of strawberry worldwide. Symptoms are characterized by wilting and stunting of plants and eventual collapse due to crown rot. In Florida, production is based on an annual plasticulture system with mulched, raised beds that are fumigated prior to planting^3^. New transplants acquired every season are the major source of inoculum for this pathogen^4^. After transplanting, daytime overhead irrigation is provided for 10 to 14 days for plant establishment, which creates a conducive environment for disease development immediately at the beginning of the season. Leather rot (LR), also caused by *P. cactorum*, affects fruit in all development stages and causes discoloration, and unpleasant taste and odor^5,6^. Because symptoms on ripped fruit are sometimes subtle, infected fruit could be picked along with healthy fruits and become a post-harvest problem, besides the direct pre-harvest losses^7^. However, epidemics of this disease are sporadic and associated with heavy rainfall events during the fruiting stage^5^.

Management of both diseases is based on the combination of resistant cultivars, cultural practices, and chemical applications^7,8^. Resistant cultivars may be available, but chemical applications have been widely used since most cultivars are mainly bred and selected based on fruit quality and yield and not for resistance to *Phytophthora*^9^. Although azoxystrobin is labeled for LR, and phosphite products for both diseases, mefenoxam is the most widely used chemical to manage PhCR and LR^10^. However, likely due to repetitive application of same chemicals in nursery and fruit production fields, *P. cactorum* isolates resistant to mefenoxam and azoxystrobin have been found in Florida strawberry fields^11,12^. In this scenario, fungicide sensitivity monitoring plays an important role in the implementation of integrated disease management programs. Currently, monitoring of *Phytophthora* spp. from strawberry for mefenoxam resistance has been done exclusively through in vitro screening, in which isolates were grown on fungicide amended and non-amended media^11,13^. These methods could be time-consuming and labor-intensive and techniques that provide rapid and accurate information about mefenoxam sensitivity in *Phytophthora* populations are needed.

Mefenoxam, is a site-specific phenylamide fungicide with systemic activity and oomycete specificity, classified as having a high risk for pathogen-resistance selection^13–15^. Several *Phytophthora* spp. resistant to mefenoxam have been reported in multiple hosts, such as *P. capsici* on cucurbits and peppers, *P. infestans* on tomato, and *P. erythroseptica* on potatoes^16–21^. In some cases, fitness penalties, such as slow growth, are associated with mefenoxam-resistant isolates ^14,15^; however, stable resistance has been reported in *P. insfestans* without fitness disadvantages^22^. It is hypothesized that mefenoxam acts on specific sites responsible for ribosomal RNA (rRNA) synthesis, which reduces mycelial growth and zoospore germination^14,18,23,24^. Because the RNA polymerases are multi-subunit complexes and topoisomerases and transcription factors could also influence their activity, the precise mefenoxam target remains unknown^22,25–27^. In *P. infestans*, for example, eight single nucleotide polymorphisms (SNPs) within the *RPA190* gene were found associated with resistance, but more recently, several candidate genes, including a homolog of yeast ribosome synthesis factor Rrp5, which is required for the processing of pre-rRNA transcripts into molecules that form ribosomes^28^, were identified in mefenoxam-resistant isolates of *P. capsici*^29^. To our knowledge, studies regarding the mechanisms, genes, and mutations involved with mefenoxam resistance in *P. cactorum* affecting strawberry have not been published.

The efficacy of mefenoxam in strawberry production has been threatened by the emergence of fungicide-resistant populations, putting the control of *Phytophthora* diseases in strawberry at risk^11,13^. Whole genome sequencing comparison of *P. cactorum* isolates representing sensitive and mefenoxam-resistant populations aiming identification of possible SNPs/mutations is crucial for comprehending the mechanisms associated with resistance. Moreover, once the candidate genes are identified, molecular tools could be developed to screen these candidate regions to differentiate sensitive and resistant populations. High-resolution melting (HRM) assay is a suitable and cost-effective method for identifying genetic variation, mutations, and SNP in DNA sequences^30^ and could be deployed to distinguish between populations of *P. cactorum* sensitive and resistant to mefenoxam. In fact, this tool has been frequently used in plant pathology to identify and differentiate pathogens and detect mutations related to fungicide resistance^31–35^.

Therefore, the main goal of this study was to determine the sequence mutations associated with mefenoxam resistance in *P. cactorum* of strawberry and differentiate sensitive and resistant populations. In the present study, we searched for mutations/SNPs in the subunits of RNA polymerase I in mefenoxam-resistant isolates and performed whole-genome sequencing of resistant and sensitive isolates to aid the identification of mutations associated with mefenoxam resistance. We further developed a high-throughput HRM diagnostic system to differentiate mefenoxam-sensitive and -resistant isolates for rapid fungicide monitoring and management recommendations.

## Results

### Identifying mutations in the RNA polymerase (*RPA190*), RNA polymerase I subunit I (RPA 1), and RNA polymerase I subunit II (RPA 2) genes

**Figure 1:**
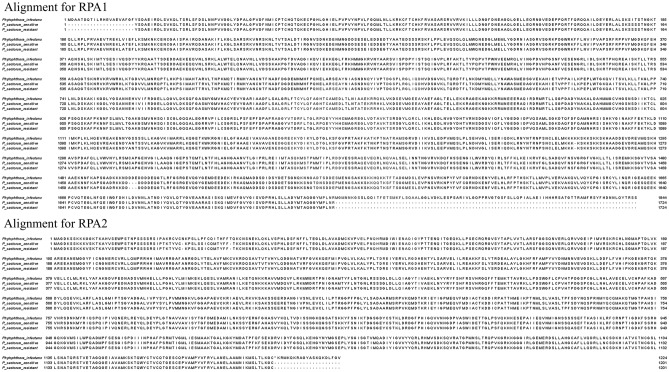
Amino acid sequences of genes RPA 1 and RPA 2, previously found in *Phytophthora infestans* conferring mefenoxam resistance (Chen et al. 2018), aligned with representative sensitive (09–100) and resistant (16–365) isolates of *P. cactorum*.

The eight mutations/SNPs responsible for amino acid changes in metalaxyl-resistant isolates of *P. infestans* were not identified in any of the *P. cactorum* resistant isolates screened in this study. Sequences of both sensitive and resistant isolates were identical for all the sites where SNPs had been previously reported in *P. infestans* resistant isolates (Table 1). After sequencing the whole RPA 1 (5540 bp) and RPA 2 (3602 bp), mutations/SNPs were not observed in any of the screened *P. cactorum* isolates resistant to mefenoxam. In fact, sequences of both sensitive and resistant isolates were identical (Fig. 1).

**Table 1:** Translation of sequences of *Phytophthora cactorum* isolates sensitive and resistant to mefenoxam for all the sites where SNPs had been previously reported in *P. infestans*.

Species	Profile	Mutation	Amino acid sequences
*P. infestans*	R	K267E	DTIRGNVSDN**K**DENMNGDDSE
*P. cactorum*	S		DTIRGNVSDK**E**DENMNGDDSE
*P. cactorum*	R		DTIRGNVSDK**E**DENMNGDDSE
*P. infestans*	R	R296H	TYAATEDSSS**R**SKFLPPLEVQ
*P. cactorum*	S		TYAATEDSSS**R**SKFLPPLEVQ
*P. cactorum*	R		TYAATEDSSS**R**SKFLPPLEVQ
*P. infestans*	R	F382Y	QNSHLSKIMT**Y**SESIVQSDYY
*P. cactorum*	S		QNSHLSKIMT**Y**SESIVQGDYY
*P. cactorum*	R		QNSHLSKIMT**Y**SESIVQGDYY
*P. infestans*	R	T443A	SSKAKPGTDV**A**QGIKQVIEKK
*P. cactorum*	S		SSKAKPGTDV**A**QGIKQVIEKK
*P. cactorum*	R		SSKAKPGTDV**A**QGIKQVIEKK
*P. infestans*	R	A597T	LHKPSIMAHT**A**RVLTNPKMQT
*P. cactorum*	S		LHKPSIMAHT**A**RVLTNPKMQT
*P. cactorum*	R		LHKPSIMAHT**A**RVLTNPKMQT
*P. infestans*	R	A871T	LLEKKRAGEK**A**GKKRRMNEEE
*P. cactorum*	S		LLEKKRAGEK**N**GKKRRMNEEE
*P. cactorum*	R		LLEKKRAGEK**N**GKKRRMNEEE
*P. infestans*	R	P980S	VPILCSGRSL**P**SFEPFDPAPR
*P. cactorum*	S		VPILCSGRSL**P**SFEPFDPAPR
*P. cactorum*	R		VPILCSGRSL**P**SFEPFDPAPR
*P. infestans*	R	V1476G	LISREMKKSG**V**TVSAAAEKNN
*P. cactorum*	S		LISREMKKSG**V**TVSAAAEKNN
*P. cactorum*	R		LISREMKKSG**V**TVSAAAEKNN

Sequences of *P. infestans* were extracted from Chen et al. 2018; Sensitive (S) and resistant (R) profiles were determined by discriminatory doses of 5 and 100 μg/ml as proposed by Marin et al. 2021.

### Identification of sequence polymorphic variants associated with mefenoxam resistance using whole genome sequencing of *P. cactorum* isolates

**Figure 2:**
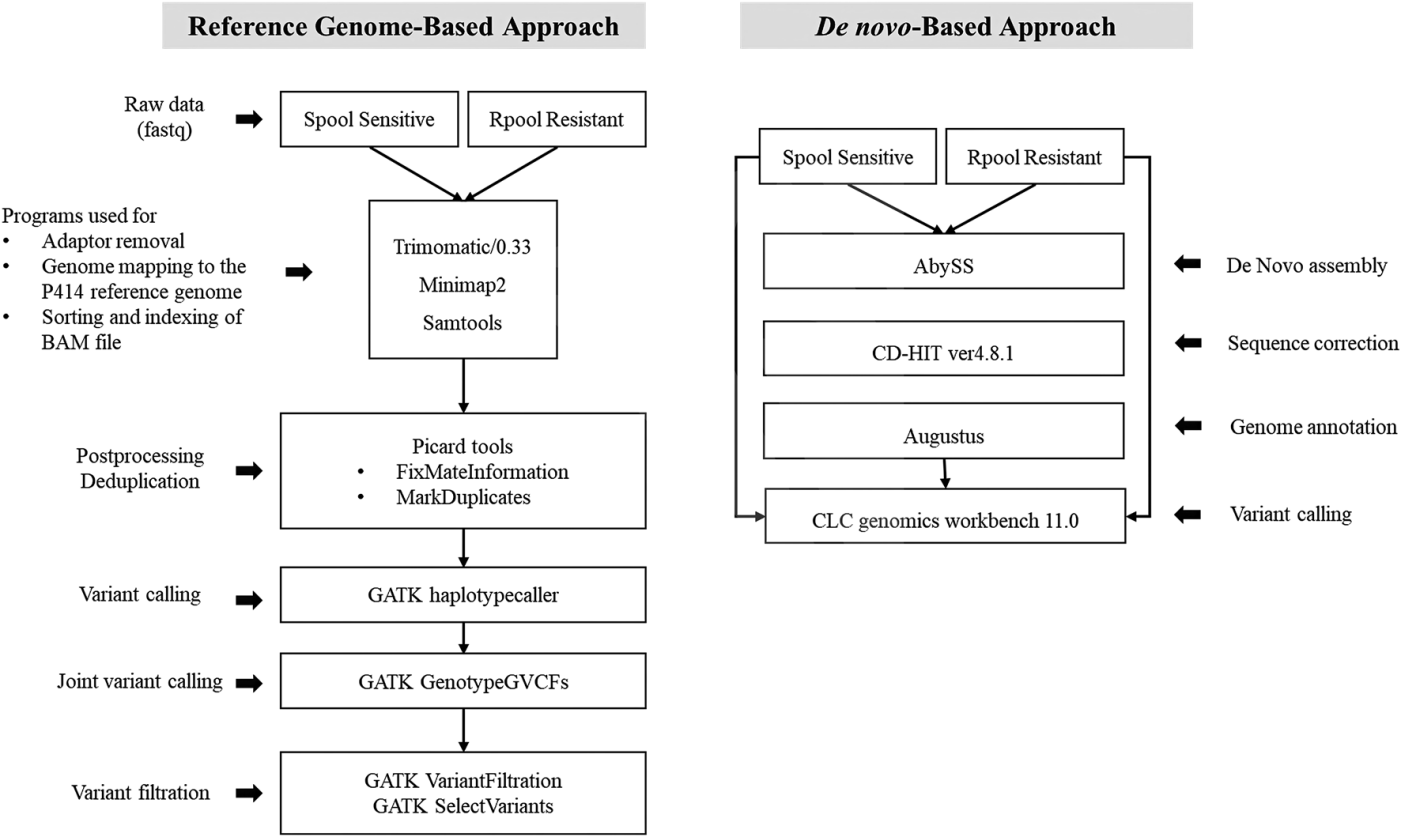
Depiction of two approaches implemented for calling sequence variants linked to the mefenoxam-resistant *P. cactorum* isolates.

Due to the lack of a high-quality chromosome-scale reference genome of *P. cactorum*, two approaches were implemented to determine the maximum number of variants related to mefenoxam resistance (Fig. 2). The basic whole-genome sequencing statistics are shown in Table 2. Using the reference genome-guided approach, after trimming and quality filtering of Illumina raw reads, 95.54 and 95.65% reads of sensitive (Spool) and resistant pool (Rpool), respectively, were mapped to the reference genome of *P. cactorum* P414^36^. A total of 1,009,563 variants were called from Spool Sensitive and Rpool Resistant sequencing libraries. The 669,815 variants were identified in the Spool library and 645,905 variants were present in the Rpool library, respectively. After filtering the variants, 80,104 SNPs and 13,665 InDels were retained. Both, Spool and Rpool genomes are available in the National Center of Biotechnology Information (NCBI), with accession numbers SRR21832435 and SRR21832436, respectively.

**Table 2:** Statistics for whole-genome sequencing of the mefenoxam-resistant strains of *Phytophthora cactorum*.

	Spool	Rpool
Total reads	74,540,816	82,532,288
Total mapped reads	71,212,819	78,944,241
Number of variants	131,176	128,796
Number of SNPs	107,883	105,612
Number of insertion	11,344	11,263
Number of deletion	11,949	11,921
Synonymous_variant	17,294	16,842
Non-synonymous_variant	23,077	22,558
Variations in exon	44,566	43,414
Genes with amino acid changes	21,610	21,149
Homozygous genotype	5,597	5,771

The resistant strains (Rpool) were compared with the mefenoxam-sensitive strains (Spool).

It is possible that sequences specifically present in the genome of strains used in this study could be missed by the P414 reference genome-guided mapping process. Thus, we conducted de novo-based assembly approach to ensure including all possible sequence variants. The high-quality reads from both Rpool Resistant and Spool Sensitive reads were merged to generate a common reference. Further, about 86.05% and 86.86% reads of Spool Sensitive reads and Rpool Resistant reads were mapped separately to the common reference, respectively. A total of 72,748 variants were identified in both Rpool Resistant and Spool sensitive groups.

**Figure 3:**
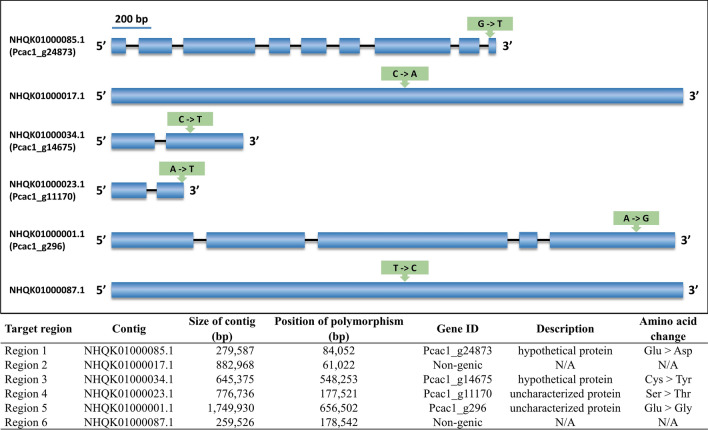
Selected SNP candidates based on the non-synonymous effect and high SNP index.

After combining all sequence variants from reference- and de novo-based approaches, we found only six regions that exhibited point mutations in the resistant mutants compared with the mefenoxam-sensitive strains (Fig. 3). Four SNPs in different chromosome regions, NHQK01000085.1 (region 1), NHQK01000034.1 (region 3), NHQK01000023.1 (region 4), and NHQK01000001.1 (region 5) were located in genic regions of four functionally unknown genes annotated as Pcac1_g24873, Pcac1_g14675, Pcac1_g11170, and Pcac1_g296. The other two SNPs located in NHQK01000017.1 and NHQK01000087.1 occurred in the non-genic regions (Fig. 3). The function of the four genes harboring mutations were predicted (Supplementary Table 3). The molecular function-GO terms for the four genes indicate their contribution to ion binding, cation binding, metal ion binding, cofactor binding, iron-sulfur cluster binding, and metal cluster binding. Biological process-GO terms indicate the functions related to small molecule and organic substance metabolic processes. The Cellular component-GO terms strongly imply that the proteins are related to the extracellular region (Supplementary Table 3). The six regions corresponding to mefenoxam resistance in *P. cactorum* mutant strains were amplified using the PCR primer sets (fragments of 202, 201, 167, 182, 162, and 192 bp) (Supplementary Table 1) obtained for each of the six regions, respectively. After sequencing the PCR products, we confirmed that the SNPs were present in the six regions identified in the whole genome analysis for all the screened resistant and sensitive isolates (Table 3).

**Table 3:** Sequencing results of PCR products from mefenoxam-sensitive and -resistant *Phytophthora cactorum* isolates.

Profile	Region	Genomic sequence
Sensitive	1	TTATGCAGGA**G**AAACGCAAGC
Resistant		TTATGCAGGA**T**AAACGCAAGC
Sensitive	2	ACAGGATCTT**C**TTGCCGTGAA
Resistant		ACAGGATCTT**A**TTGCCGTGAA
Sensitive	3	GATGTCATTG**C**AGAAGAAGAA
Resistant		GATGTCATTG**T**AGAAGAAGAA
Sensitive	4	GGTGCTACCG**T**CGTCCTCGCT
Resistant		GGTGCTACCG**A**CGTCCTCGCT
Sensitive	5	CACCAACACG**A**GAGTCTAGAA
Resistant		CACCAACACG**G**GAGTCTAGAA
Sensitive	6	ACTCTGAAAG**T**CTAAACACCA
Resistant		ACTCTGAAAG**C**CTAAACACCA

### High-resolution melting (HRM) assay for detection of SNP mutations

**Figure 4:**
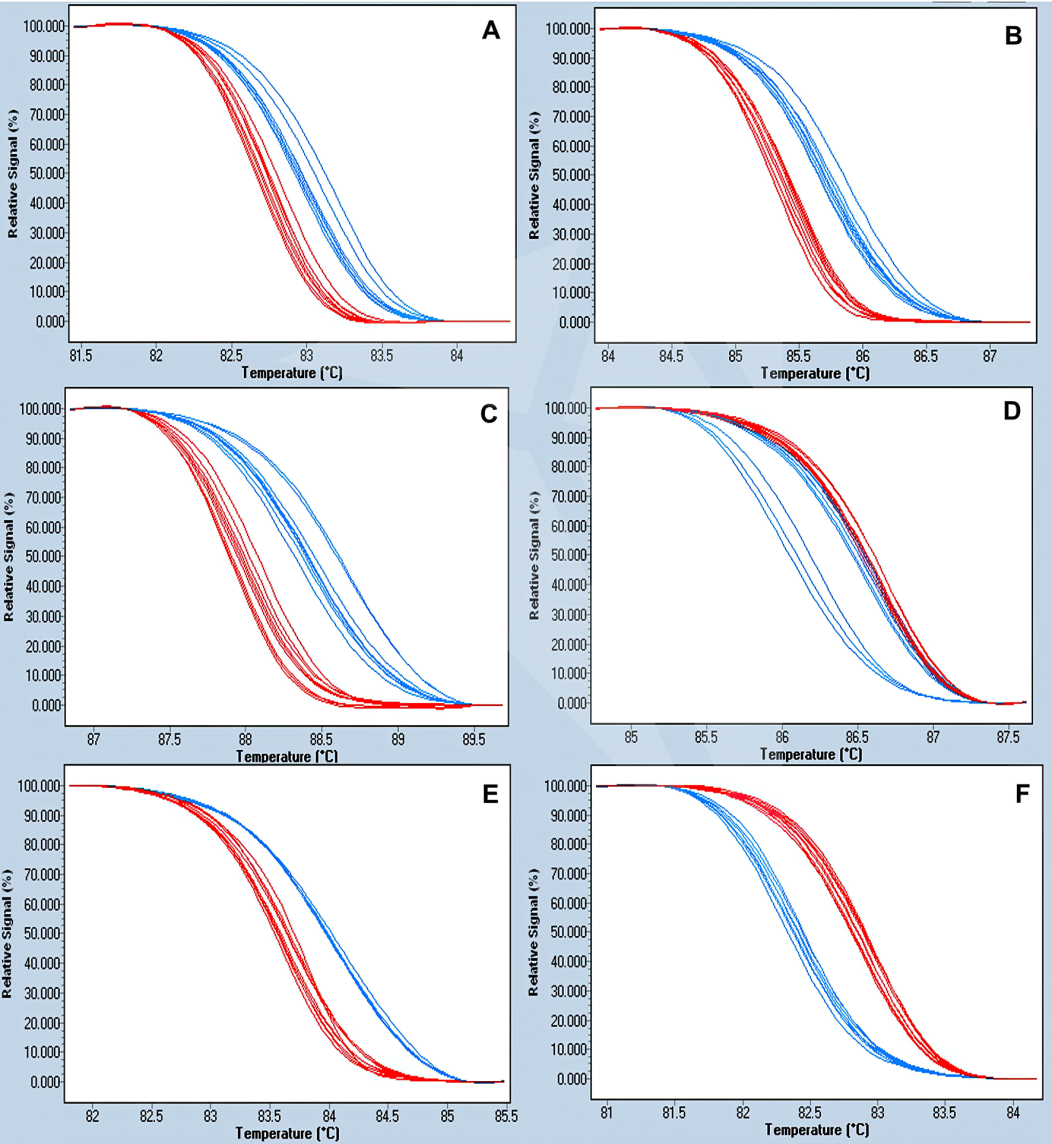
Normalized high-resolution melting (HRM) curves of regions 1 (**A**), 2 (**B**), 3 (**C**), 4 (**D**), 5 (**E**), and 6 (**F**) for the identification and differentiation of mefenoxam-sensitive (blue) and -resistant (red) isolates of *P. cactorum* of strawberry.

**Figure 5:**
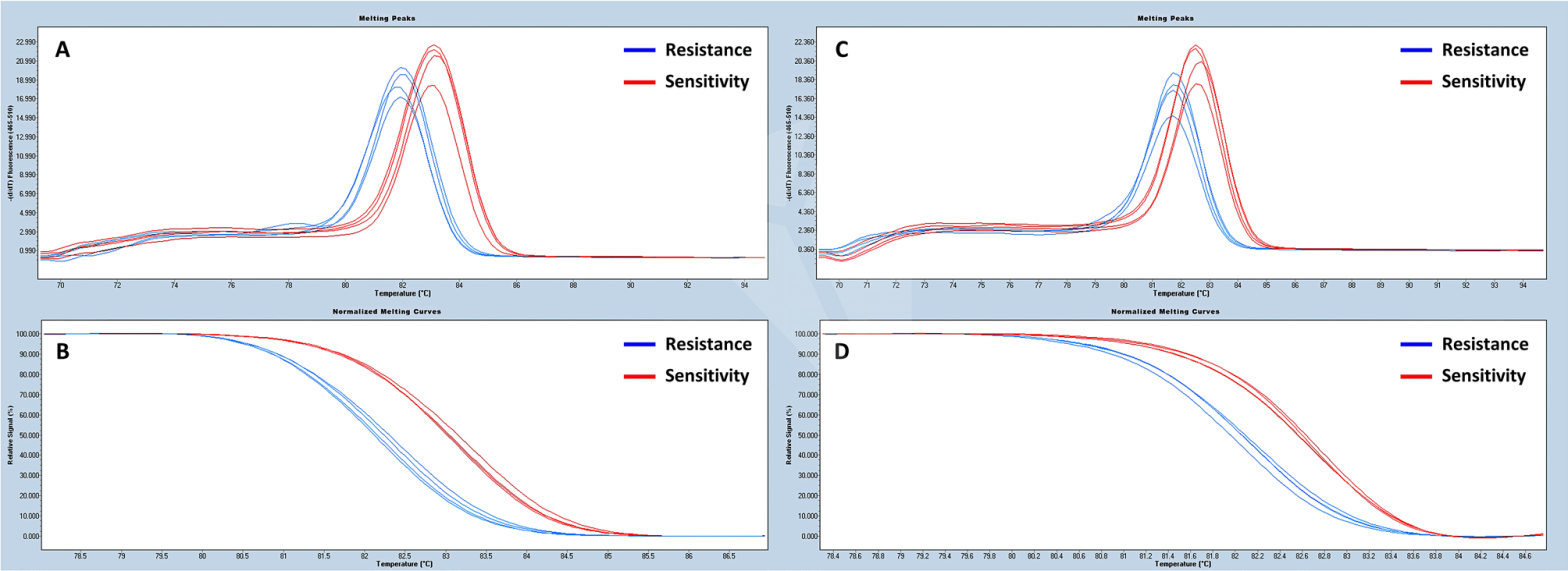
HRM analysis of R3-1 and R2-1 HRM markers with clean DNA extracts of 28 resistant and 12 sensitive isolates. (**A,B**) Melt curve genotyping and gene scanning analysis of the R3-1 marker. (**C,D**) Melt curve genotyping and gene scanning analysis of the R2-1 marker.

**Figure 6:**
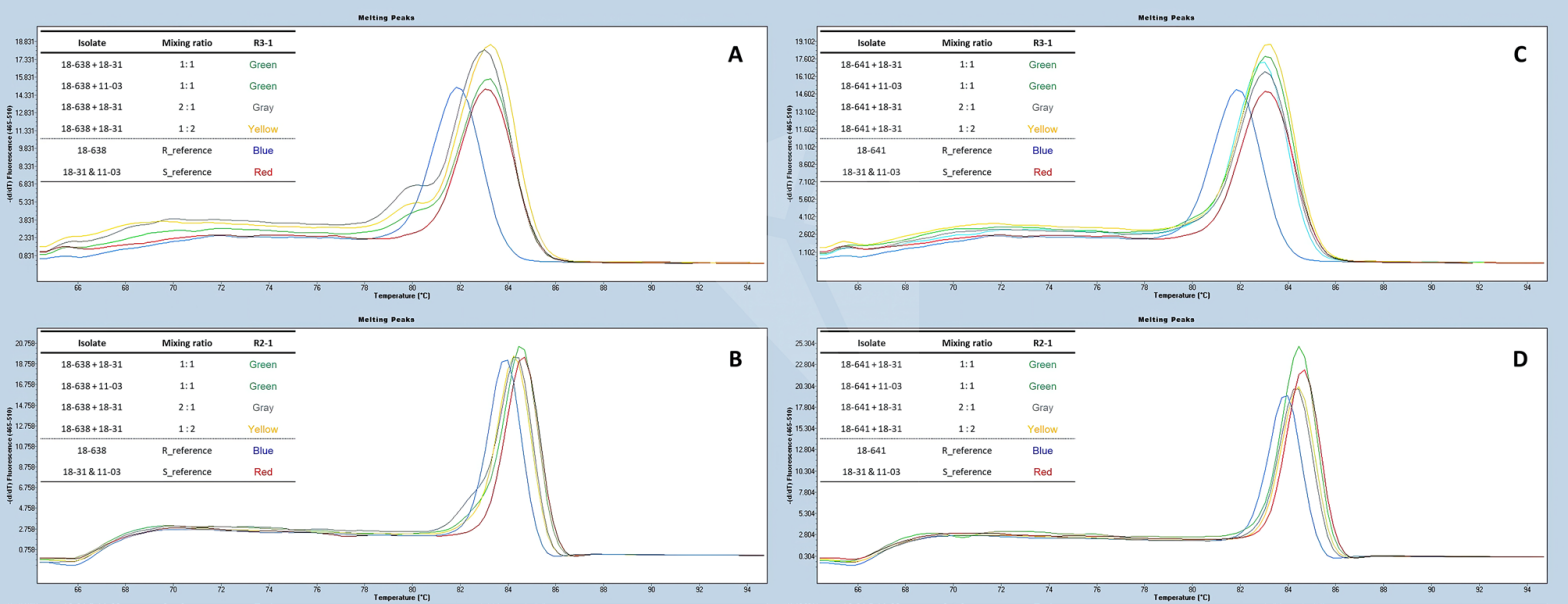
HRM analysis on a mix of resistant and sensitive isolates in the clean DNA condition using R3-1 and R2-1 HRM markers. (**A,B**) HRM results of the combinations of resistant isolate, 18-638, and the two sensitive isolates, 11-03 and 18-31, in different ratios with R3-1 and R2-1 markers, respectively. (**C,D**) HRM results of the combinations of resistant isolate, 18-641, and the two sensitive isolates, 18-31 and 11-03, in different ratios with R3-1 and R2-1 markers, respectively.

HRM markers were designed to amplify the target mutant regions of the genome of *P. cactorum* for the differentiation of mefenoxam-sensitive and -resistant isolates. Analysis of the HRM curves generated two distinct melting profiles for most of the regions except for region 4, allowing the accurate differentiation of sensitive and resistant isolates (Fig. 4 and Supplementary Fig. 1). Sequences were identical within mefenoxam-profiles, whereas they were different between sensitive and resistant groups. To better differentiate the two populations, the HRM markers R3-1 from region 3 and R2-1 from region 2 were developed by targeting SNP (C/T) in Pcac1_g14675 and SNP (C/A) in the contig NHQK01000017.1 (Fig. 3). The HRM results showed that re-designed HRM markers targeting regions 3 and 2 greatly improved the differentiation between mefenoxam-sensitive and -resistant isolates of *P. cactorum* (Figs. 5 and 6).

In the primer test using pure DNA, HRM analysis was performed with two HRM markers, R3-1 and R2-1, in a total of 40 isolates consisting of 28 resistance and 12 sensitive isolates, respectively. The 28 resistant and 12 sensitive representative isolates were clearly divided into resistance (blue) and susceptibility (red) curve patterns in the HRM analysis, respectively (Fig. 5 and Supplementary Table 2). For the rapid detection of mefenoxam resistance, two HRM markers, R3-1 and R2-1, were also examined for HRM curve patterns between resistant and sensitive isolates in the crude DNA extracts. In the HRM analysis, out of 16 tested samples, five sensitive-phenotype isolates showed a sensitive curve pattern (red), while the remaining 11 resistant-phenotype isolates had a distinct curve pattern (blue), resembling the separation whenever pure DNA was used (Fig. 6).

HRM analysis on a mix of resistant and sensitive isolates in the clean DNA condition was also performed. When one resistant isolate, 18-638, and two sensitive isolates, 18-31 and 11-03, were mixed in ratios of 1:1, 1:2, and 2:1, respectively, HRM results of R3-1 and R2-1 markers revealed that all mixtures showed a heterozygous HRM pattern (Fig. 6A, B). However, when one resistant extract, 18-641, and two sensitive mixtures, 18-31 and 11-03, were mixed at the same ratios, 1:1, 1:2, and 2:1, a sensitivity HRM pattern with R3-1 and R2-1 markers was observed (Fig. 6C, D).

## Discussion

Mefenoxam has been considered the gold standard to control strawberry diseases caused by *Phytophthora* spp. However, the emergence of resistant isolates could threaten the use of this chemical in strawberry commercial fields. In this study, we have identified variations in unknown genes contributing to mefenoxam resistance and developed a high-throughput HRM assay that could rapidly detect mefenoxam-resistant isolates to aid timely management recommendations for strawberry growers and nurseries.

Mefenoxam-resistant isolates were first observed in Florida during the 2015–2016 strawberry season, but sensitive isolates are still predominant within the population. Fortunately, resistance is not widespread throughout strawberry nurseries, but limited to nurseries in North Carolina state^11^. The registration of additional fungicides with different modes of action would allow fruit and nursery growers to alternate products to reduce fungicide resistance risk^37^. However, currently, chemical options to manage PhCR are limited to mefenoxam and phosphite products^10^. Since mefenoxam is considered a premium product, repetitive applications across nursery and fruit production fields may have selected resistant isolates. Although studies aiming to evaluate fitness penalties on *P. cactorum* mefenoxam-resistant isolates were not carried out since resistance was found, most of the growers that acquired transplants from the affected North Carolina nurseries experienced failure of mefenoxam control, suggesting that the resistant population had been established in those nurseries. Fitness penalties on resistant isolates, such as slower growth, have been reported on some *Phytophthora* species^14^, which could be a factor limiting the widespread resistance. However, stable resistance without fitness disadvantages circumventing the fungicide effect of mefenoxam has been reported in *P. infestans*^22^.

Previous studies hypothesized that the efficacy of mefenoxam is related to the binding and inhibition of specific sites responsible for ribosomal RNA (sRNA) synthesis, implying the reduction in mycelial growth and zoospore germination^14,18,23,24^. However, because RNA polymerases are multi-subunit complexes and topoisomerases and transcription factors could also influence their activity, the precise mefenoxam target remains unknown^22,25–27^. In *P. insfestans*, resistant isolates were conditioned by variation in the RNA polymerase gene; however, the inheritance profile from crossing a sensitive and resistant isolate suggested the involvement of more than one locus^22,38^. Controversially to the studies carried out with *P. infestans*, variations within the RPA 1 and RPA 2 genes conferring resistance to mefenoxam were not identified on *P. cactorum* isolates, but six SNPs associated with resistance were identified after the whole genome sequencing of mutant isolates. Similar findings were reported in *P. capsici* mefenoxam-resistant isolates, where the major SNPs conferring resistance were not within the RNA polymerase genes, but in a homolog of yeast protein Rrp5 gene required for processing of pre-rRNA transcripts into the cleaved molecule that forms the ribosome^28,29^ Interestingly, after the discovery of these six SNPs, screening of all resistant isolates revealed that all the SNPs occurred simultaneously. A definitive test to determine whether all alleles are needed to confer resistance to mefenoxam, or which one has a major effect, using genetic transformation to study the interaction and effect of each locus alone would be necessary. Moreover, the detection of all the SNPs in all the resistant isolates could be due to clonal reproduction, since the isolates originated from the same nursery source in North Carolina. Therefore, there is a chance the SNPs found in this study conferring mefenoxam resistance, could vary in *P. cactorum* isolates selected for resistance in other nurseries, growing systems, or crops.

In this study, based on functional annotation, the genes harboring mutations associated with resistance to mefenoxam in *P. cactorum* were involved in ion, cation, metal, cofactor, iron-sulfur cluster, and metal cluster binding. These are process related to metabolism of substances, which based on component-GO strongly indicate the proteins are related to the extracellular region. Metal ions can be toxic to fungal cells due to their ability to disrupt cellular processes and structures through the generation of reactive oxygen species (ROS), and interfere with enzyme activity by binding to the active site of enzymes that inhibit their functions. Mutations in genes involved in metal ion binding can result in decreased binding affinity of proteins for metal ions, decreasing the effectiveness of the fungicide. This could lead to the development of resistance in fungal pathogens ^39–41^. Minor effects of several other genes have also been reported to contribute to resistance, such as genes related to efflux pumps and detoxification as ATP binding cassette (ABC) transporters and cytochrome P450 proteins^14,42,43^. Moreover, some ABC proteins and metal ion binding proteins could acted as transmembrane transporters to transfer protons crossing the inner membrane of mitochondria thus compensate for the disrupted proton gradient. All these functional groups may be the primary step of SYP-14288 function and then caused the downstream intricate biological reaction^44^. This is the first study exploring the mechanism of resistance to mefenoxam in *P. cactorum.*

The practical application of determining the SNPs associated with the mefenoxam resistance would be the development of DNA markers for rapid differentiation of sensitive and resistant isolates. Screening isolates for fungicide resistance through in vitro tests is widely used and the use of discriminatory doses for testing mefenoxam was proposed^11^. However, this method is timing-consuming which can delay management recommendations. In this study, an HRM assay is proposed to speed up the process of distinguishing sensitive and resistant populations of *P. cactorum* to mefenoxam. Primers were designed for all the SNPs identified, and HRM curves generated two distinct melting profiles for most of the regions except for region 4. The issue with region 4 is that the replacement of T (thymine) to A (adenosine), or vice-versa does not change the number of hydrogen bonds (n = 2), which limits the detection of the polymorphism “T/A” in the HRM analysis. The pair of primers R3-1F/R3-1R and R2-1F/R2-1R were suitable to differentiate both sensitive and resistant profiles using clean and crude DNA extraction. However, if there is a mixture of sensitive and resistant populations, shifts in the melting curves could be expected for both primers. The use of crude extraction of symptomatic strawberry crown tissues was proposed by Wang et al.^35^ to diagnose *Colletotrichum* spp., *Macrophomina phaseolina*, and *Phytophthora* spp., and the same extracts could be used to detect mefenoxam resistance if samples are positive for *P. cactorum*, which could faster management recommendations to the strawberry industry and growers.

Although strawberry cultivars resistant to PhCR are available, susceptible ones are widely grown based on their fruit quality and yield. Therefore, the application of chemicals for disease management will continue to provide security for crop production. The determination of genomic regions conditioning fungicide resistance could offer the possibility of accurately monitoring the pathogen population to guide disease control strategies. In this study, we have identified sequence variation in unknown genes that were correlated with the mefenoxam-resistant profile of *P. cactorum* isolates from strawberry. Furthermore, an HRM assay was designed to be implemented in diagnostic clinics to improve and faster management recommendations. Our findings may contribute to better understanding of the mechanisms of action of mefenoxam in oomycetes as well as contribute to the sustainable use of this product by avoiding its application when resistance is detected.

## Materials and methods

### Collection and storage of *P. cactorum* isolates

Isolates of *P. cactorum* were collected between 1997 to 2020 from strawberry samples showing PhCR and LR symptoms received by the Diagnostic Clinic at the University of Florida Gulf Coast Research and Education Center (UF-GCREC). However, resistance to mefenoxam was found only in some isolates collected after the 2015–16 Florida strawberry season. Isolates were purified by hyphal-tipping on cornmeal agar amended with pimaricin, ampicillin, rifampicin, and pentachloronitrobenzene (P_5_ARP^45^), and were identified at the species level using the high-resolution melting analysis developed by Wang et al.^35^ using genomic DNA and the set of primers Ph29-F and Ph29-R as described by Ratti et al.^34^. Isolates were transferred to 20% V8 media^46^ containing 0.03 g/l of β-sitosterol for 7 to 10 days, then stored in deionized water at room temperature (~ 24 °C). In total, 54 sensitive and 31 resistant isolates were hyphal-tipped and molecularly screened for mefenoxam sensitivity. Cultures were grown in P_5_ARP and transferred to 20% V8 medium amended with 0.03 g/L β-sitosterol for 7–10 days at 25 ºC. Mycelial plugs (6-mm-diameter) were used for storage in sterile water at 25 °C and in 30% glycerol at −80 °C in the culture collection of the Strawberry Pathology laboratory at the UF-GCREC. The mefenoxam resistant isolates did not show any growth inhibition on clarified 20% V8 media amended with 5 and 100 mg/µl of mefenoxam, according to Marin et al.^11^ (Supplementary Fig. 2).

### DNA extraction

Isolates were grown on 20% V8 media for 5–7 days and mycelial plugs from actively growing margins of the cultures were collected for DNA extraction using the FastDNA Kit (MP Biomedicals), following the manufacturer’s protocol. The quality and quantity of DNA samples were determined using a NanoDrop 8000 spectrophotometer (ThermoFisher Scientific). DNA samples with a 260/280 ratio between 1.7 and 1.9 and a 260/230 ratio greater than 2.0 were diluted to a final concentration of 10 ng/μL and stored at −20 °C.

For the *P. cactorum* whole genome sequencing, a protocol for plant DNA extraction modified from Keb-Llanes et al.^47^ by Integrated DNA Technologies (IDT) was used for the pathogen DNA extraction. Colonies were grown on 10% V8 broth for four days, and then mycelia were washed with sterile deionized water, flash frozen in liquid nitrogen, and ground using a mortar and pestle. Subsequent extraction steps followed the protocol above mentioned. DNA concentration was verified using a NanoDrop 8000 spectrophotometer (ThermoFisher Scientific). DNA quality was determined using the Invitrogen Qubit Fluorometer (Invitrogen Life Technologies, ThermoFisher Scientific), according to the manufacturer’s protocol and concentration was adjusted to 50 ng/μL.

### Detecting mutations/single nucleotide polymorphisms (SNPs) in the RNA polymerase (*RPA190*) gene reported in *P. infestans*

A total of 35 single nucleotide polymorphisms (SNPs) in the *RPA190* gene (5433 bp) encoding the large subunit of RNA polymerase I in metalaxyl-resistant isolates of *P. infestans* were identified by Chen et al.^38^. Eight SNPs caused amino acid mutations associated with metalaxyl resistance in resistant isolates compared with sensitive isolates: K267E, R296H, F382Y, T443A, A597T, A871T, P980S, and V1476G^38^. Sequences flanking the mutations from *P. infestans* were compared with the *P. cactorum* whole genome sequence published by Armitage et al.^48^. Based on the regions containing the mutations, five sets of primers were developed: *RPA190*-F1/R1 to *RPA190*-F5/R5 (Supplementary Table 1), using the IDT-PrimerQuest Tool (https://www.idtdna.com/PrimerQuest/Home/Index). Ten sensitive and ten resistant *P. cactorum* isolate were selected. PCR conditions were as follows: 15 µL of 2× AccuStart II PCR ToughMix (Quantabio; Gaithersburg, Maryland—USA), 1.5 µL of each forward and reverse primers (10 µM), 11 µL of molecular water, and 1 µL of diluted DNA (10 ng/μL), with a total volume of 30 µL. Amplifications were performed according to the following conditions: initial DNA denaturation at 95 °C for 4 min; followed by 32 cycles of denaturation at 95 °C for 30 s, annealing at 57 °C for 30 s, and extension at 72 °C for 3.5 min, and a final extension at 72 °C for 10 min. PCR products were visualized under UV light in a 1% agarose gel in 1× Tris–acetate-EDTA buffer stained with (Biotium) and sent for purification and sequencing in both directions at Genewiz Inc. (South Plainfield, NJ). Sequences were aligned using Geneious (version 11.1.4) and MEGA (version 7.0.20) software programs and the five amplified regions of sensitive and resistant isolates were compared separately.

### Identifying mutations/single nucleotide polymorphisms (SNPs) in the RNA polymerase I subunit I (RPA 1) and II (RPA 2)

Based on the *P. cactorum* whole genome sequence published by Armitage et al. (2018), various primers were designed for amplification and sequencing of the whole RNA polymerase I subunit I (RPA 1) and subunit II (RPA 2) genes. In order to locate both genes within the genome, sequences from *P. infestans* XM_002906849 and XM_002907301 were used for RPA 1 and RPA 2, respectively. Six sets of primers were developed for RPA 1 (RPA1-F1/R1 to RPA1-F6/R6), and four for RPA 2 (RPA2-F1/R1 to RPA2-F4/R4) (Supplementary Table 1). PCR conditions, product visualization, and sequencing were performed for five sensitive and five resistant isolates as described in the previous section. Sequences of RPA 1 and RPA 2 of one sensitive and one resistant isolate were submitted to GenBank (accession numbers: OM273467–OM273470).

### Whole genome sequencing and SNP variant calling

One pool containing 18 sensitive isolates and another pool with 18 resistant isolates were submitted for whole genome sequencing by Novogene (Chula Vista, CA) with Illumina Platform PE150, Q30 ≥ 80%. Illumina raw reads adapter trimming and quality filter were performed using CLC genomics workbench 11.0 (https://www.qiagenbioinformatics.com/). Two approaches were implemented to identify sequence variants related to mefenoxam resistance.

#### Variant calling by mapping to reference-based approach

High-quality trimmed reads from mefenoxam-sensitive pooled reads (Spool Sensitive) and mefenoxam-resistant pooled reads (Rpool Resistant) were separately mapped to the two reference genomes of *P. cactorum*^36,49^. Raw reads were trimmed using Trimmomatic v.0.32^50^. Trimmed reads were mapped to the whole genome sequence of ‘P414′^36^ (https://www.ncbi.nlm.nih.gov/bioproject/PRJNA383548) using Minimap2 with default parameters^51^. The file with sequence alignment/map (SAM) format was converted into a binary alignment map (BAM) format with SAMtools v.1.12^52^. DNA variants including SNPs and insertion/deletion (InDel) were called using a genome analysis toolkit (GATK^53^) according to the manuals with default parameters. Variants were filtered using the following VCF parameters: QD > 2.0, FS > 60, ReadPosRankSum < −8.0, and MQRankSum < −12.5. Alternatively, the low frequency variant caller program of CLC genomics workbench 11.0 was also implemented to call variants from Spool Sensitive and Rpool Resistant reads. Variant filtration was performed by eliminating the common SNPs between Spool Sensitive and Rpool Resistant groups to select only the non-synonymous SNPs. The SNPs with only a high SNP index (> 80%) were considered for further validation.

#### De novo-based approach for calling variants

For this approach, high-quality reads from both “Rpool Resistant” and “Spool Sensitive” were merged for de novo assembly. The software AbySS 2.1.1^54^ was chosen for de novo assembly because of its better performance against short reads. Strict parameters were used for de novo assembly and the contigs were made non-redundant using software CD-HIT v4.8.1^55^. The contigs having a length less than 2 kb were discarded and not considered for further analysis. Software Augustus v2.5.5^56^ was used for gene prediction. Further, reads from both Rpool Resistant and Spool Sensitive were mapped to the final de novo assembled contigs separately for variant calling. The low frequency variant caller program of CLC genomics workbench 11.0 was used to call variants from Spool Sensitive and Rpool Resistant reads separately. Common variants were removed between Spool Sensitive and Rpool Resistant groups. Only non-synonymous SNPs with a high SNP index were selected for further analysis.

#### Functional annotations of gene-containing point mutations

The function of the four genes harboring mutations was predicted using DeepFRI, a method based on graph convolutional networks for annotating proteins and identifying functional regions in proteins (Gligorijević et al. 2021; https://beta.deepfri.flatironinstitute.org/).

### Sequencing the genomic regions associated with mefenoxam resistance

Based on the flanking sequence contigs (200 bp) originated from the whole genome sequencing of a pool of sensitive and resistant *P. cactorum* isolates, seven regions containing the SNPs possibly associated with mefenoxam resistance were amplified through PCR and sequenced. Six sets of primers were developed to amplify the seven regions and designated as 1, 2, 3, 4, 5, and 6: Rdmyl1 F/R to Rdmyl6 F/R (Supplementary Table 1). PCR conditions, product visualization, and sequencing were performed as described in the other PCR sections.

### High-resolution melting (HRM) assay for detection of SNP mutations

Based on the whole genome sequencing results, markers were designed to amplify the six flanking sequence regions containing the SNPs/mutations that could potentially be associated with mefenoxam resistance in *P. cactorum* using the IDT-PrimerQuest Tool (https://www.idtdna.com/PrimerQuest/Home/Index). Different sets of markers HRM 1F/1R, HRM 3F/3R, HRM 5F/5R, S1F/S1R, R4F/R4R, and R6F/R6R were used to amplify the six respective regions of 54 sensitive and 31 resistant isolates (Supplementary Table 1). PCR and HRM were performed in a Roche LightCycler 480 Instrument II (Roche Diagnostics, Indianapolis, IN). Reactions had a total volume of 10 μL in a 384-well plate (TempPlate 384-well Full-Skirt PCR plate, White; USA Scientific, Ocala, FL) containing 5 µL of 2× AccuStart II PCR ToughMix (Quantabio; Gaithersburg, Maryland—USA), 0.5 µL of each forward and reverse primers (10 µM), 0.5 µL of LCGreen Plus dye (BioFire Defense, Salt Lake City, UT), 2.5 µL of molecular water, and 1 µL of diluted DNA (10 ng/μL). PCR amplification began with an initial denaturation at 95 °C for 3 min (ramp rate: 4.8 °C/s), followed by 35 cycles of 95 °C for 20 s (ramp rate: 4.8 °C/s), 61 °C for 30 s (ramp rate: 2.5 °C/s), and 72 °C for 30 s (ramp rate: 4.8 °C/s). For post-PCR analysis, samples were subjected to the following HRM parameters: 95 °C for 1 min (ramp rate: 4.8 °C/s), 40 °C for 1 min (ramp rate: 2.5 °C/s), 65 °C for 1 s (ramp rate: 4.8 °C/s), and then a continuous fluorescence reading from 65 to 95 °C (ramp rate: 0.02 °C/s, 25 acquisitions/°C), with a final cooling step at 40 °C for 30 s (ramp rate: 2.5 °C/s). Melt curve genotyping and gene scanning analysis were performed using the LightCycler 480 software (version 1.5.1.62). To generate the normalized melting curves, pre-melt and post-melt temperature settings were adjusted according to each region analyzed, and the temperature threshold was set to 0 °C. Experiments were conducted in duplicate with three replications per sample.

### Rapid detection of SNP mutations associated with mefenoxam resistance directly from infected strawberry crowns

To develop HRM markers with more precise melting curve patterns between resistance and sensitive isolates, sequence variations present in the region 3 (NHQK01000034.1) and region 2 (NHQK01000017.1) containing the SNPs/mutations that could potentially be associated with mefenoxam resistance in *P. cactorum* were used to develop functional markers (Fig. 3). Primers targeting regions 2 and 3 were efficient in differentiating sensitive from resistant phenotypes and did not have much failure of amplification when testing a high number of isolates (*data not shown*). Two primer sets, R3-1 and R2-1, were designed from the polymorphic sequences of region 3 and region 2, respectively, using the IDT PrimerQuest tool (https://www.idtdna.com/PrimerQuest/Home/Index) (Supplementary Table 1). A total of 40 isolates (28 resistant and 12 sensitive isolates) were used for the primer design test, and a total of 16 crude DNA extracts from symptomatic crowns inoculated with *P. cactorum* isolates (11 resistant and five sensitive isolates) were used for marker validation (Supplementary Table 2). For the crude DNA extraction of infected strawberry crowns, plants of Sensation ‘Florida 127’ were inoculated by dipping the roots in a zoospore suspension of each isolate separately (10^4^ zoospores/ml) and potted in the greenhouse. Once symptoms of PhCR were observed, the crowns were opened, and symptomatic tissue was used for the crude DNA extraction following the protocol published by Wang et al.^35^. The two primers, R3-1 and R2-1, were also tested in a mixture of resistant and sensitive isolates of the purified DNA. Two resistant and two sensitive isolates diluted at a concentration of 20 ng/μl were used, respectively. The combinations of mixtures were pooled together with resistant and sensitive isolates in ratios of 1:1, 1:2, and 2:1, respectively. PCR reactions were prepared as mentioned above. The PCR and HRM analysis were performed in a LightCycler 480 system II using a program consisting of an initial denaturation at 95 °C for 5 min; 45 cycles of denaturation at 95 °C for 10 s, annealing at 62 °C for 10 s, and extension at 72 °C for 20 s. After PCR amplification, the samples were heated to 95 °C for 1 min and cooled to 40 °C for 1 min. Melting curves were obtained by melting over the desired range (60–95 °C) at a rate of 50 acquisitions per 1 °C. Melting data were analyzed using the Melt Curve Genotyping and Gene Scanning Software (Roche Life Science, Germany). Analysis of HRM variants was based on differences in the shape of the melting curves and melting temperature (Tm) values.

### Complies with international, national and/or institutional guidelines

Experimental research methods used in this study complies with relevant institutional, national, and international guidelines and legislation.

## Supplementary Information

Supplementary Information.

## Data Availability

The datasets generated and/or analyzed during the current study are available in the GenBank (accession numbers: OM273467–OM273470), and NCBI (accession numbers: SRR21832435 and SRR21832436). Correspondence and requests for materials should be addressed to N.P. and S.L. Reprints and permissions information is available at https://www.nature.com/reprints.
