# Supplementary material for: Identification of sequence mutations in Phytophthora cactorum genome associated with mefenoxam resistance and development of a molecular assay for the mutant detection in strawberry (F. × ananassa)
Source: Sci Rep. 2023 May 6;13:7385. doi: 10.1038/s41598-023-34271-z (PMC10164155; doi:10.1038/s41598-023-34271-z)
Supplement: Supplementary file 1 — Supplementary Information. [file 41598_2023_34271_MOESM1_ESM.docx]

**Identification of sequence mutations in *Phytophthora cactorum* genome associated with mefenoxam resistance and development of a molecular assay for the mutant detection in strawberry (*F.* ×*ananassa*)**

**Marcus V. Marin^1,2^, Juliana S. Baggio^2^, Youngjae Oh^2^, Hyeondae Han^2^, Saket Chandra^2^, Nan-Yi Wang^2^, Seonghee Lee^*2,3^,** and **Natalia A. Peres^*1,2^.**

^1^ Department of Plant Pathology, University of Florida, Gainesville, FL 32611, U.S.A.

^2^ Gulf Coast Research and Education Center, University of Florida, Wimauma FL 33598, U.S.A.

^3^ Horticultural Science Department, University of Florida, Gainesville, FL 32611, U.S.A.

^*^Corresponding authors: Natalia A. Peres ([nperes@ufl.edu](mailto:nperes@ufl.edu)) and Seonghee Lee ([seonghee105@ufl.edu](mailto:seonghee105@ufl.edu))

Supplementary Table 1. List of primers developed for this study.

| **Primer** | **Sequences 5’->3’** | **Amplicon (bp)*** | **Assay** |
| --- | --- | --- | --- |
| RPA190-F1 | TCAGACAGGATGCCAGTGC | 145 | RNA polymerase (RPA190) |
| RPA190-R1 | TTGTCGCCCATGAAAACAGG |  |  |
| RPA190-F2 | TTCCTGAACGTGATTCCCGT | 115 | RNA polymerase (RPA190) |
| RPA190-R2 | GTACGGATCAGGAGAGATAACC |  |  |
| RPA190-F3 | GCGAGGCTATCAGTAAGACG | 102 | RNA polymerase (RPA190) |
| RPA190-R3 | TTGTAAGCCTCCGAACGC |  |  |
| RPA190-F4 | GAGGCTTACGCTGAGTTTGC | 170 | RNA polymerase (RPA190) |
| RPA190-R4 | ACAAGACCTTCACGACCAGCCATA |  |  |
| RPA190-F5 | GAGCAGCGTCTGAACCAGGT | 141 | RNA polymerase (RPA190) |
| RPA190-R5 | GAATCTTCTCGTCCTCCTCATC |  |  |
| RPA1-F1 | CGTCCAATGACAACCAGTACA | 1119 | RNA polymerase (RPA I) |
| RPA1-R1 | CTTCACGACCAGCCATACAA |  |  |
| RPA1-F2 | CCACAGACCAGACCATTCTT | 1170 | RNA polymerase (RPA I) |
| RPA1-R2 | GCTTGTAGTAGTCTCCCTGAAC |  |  |
| RPA1-F3 | ACGGATCCAACAGTGACATTAT | 1188 | RNA polymerase (RPA I) |
| RPA1-R3 | AGTAGTTCACCCACACAAGAAG |  |  |
| RPA1-F4 | GGTTTGATTTCGGAGCGTATTC | 1017 | RNA polymerase (RPA I) |
| RPA1-R4 | AGATTTCGTTTGCTGCTTCTTC |  |  |
| RPA1-F5 | AGTTCTTCCTGAACGTGATTCC | 1076 | RNA polymerase (RPA I) |
| RPA1-R5 | CACACCAGCATCTTCCATACT |  |  |
| RPA1-F6 | CAGATCCGCCTGACTTTCTT | 1016 | RNA polymerase (RPA I) |
| RPA1-R6 | ATGGACGTCTCGAACGTAATC |  |  |
| RPA2-F1 | CGAAGAAGAAGGTCAGCAAGA | 1024 | RNA polymerase (RPA II) |
| RPA2-R1 | GTACACATAAGGCATGGCAAAC |  |  |
| RPA2-F2 | GGACGCTATGATCCTCAACAA | 1189 | RNA polymerase (RPA II) |
| RPA2-R2 | GCATCCCTTCAGAGTCAAAGA |  |  |
| RPA2-F3 | GGTGTCAAGAAGCAGGAGTT | 1011 | RNA polymerase (RPA II) |
| RPA2-R3 | CATAACAGGCAGATACGAGTACG |  |  |
| RPA2-F4 | TGGAATGTGAACTGGTGTGT | 1135 | RNA polymerase (RPA II) |
| RPA2-R4 | CAGAACCGGAGCTTGATACTC |  |  |
| Rdmyl1 F | ACCAGCGGAAATCACTGC | 202 | Sequencing regions identified in the whole genome analysis |
| Rdmyl1 R | GACAGGCAACCGCTCAA |  |  |
| Rdmyl2 F | GGACTTGACGCCTAGCTT | 201 | Sequencing regions identified in the whole genome analysis |
| Rdmyl2 R | TTGAGCACATACACTGTTGTTT |  |  |
| Rdmyl3 F | CCAGCAACGTGGCAATTT | 167 | Sequencing regions identified in the whole genome analysis |
| Rdmyl3 R | GTTCCTGACCCGTCCTTT |  |  |
| Rdmyl4 F | GCGTATACATCTCACTGTGGTTC | 182 | Sequencing regions identified in the whole genome analysis |
| Rdmyl4 R | GTGGTAACGCATTCTGACTATGT |  |  |
| Rdmyl5 F | CGAGGAACTATCAGTGGATCTT | 162 | Sequencing regions identified in the whole genome analysis |
| Rdmyl5 R | GCGTAAGCTTCCAGCTC |  |  |
| Rdmyl6 F | GCTCCGCAATTCGAAACAG | 192 | Sequencing regions identified in the whole genome analysis |
| Rdmyl6 R | GATTTCTCCATAGTCCGAGTACAA |  |  |
| HRM 1F | CACCTCCTGCACTTTGGTAT | 127 | HRM – Region 1 |
| HRM 1R | CCGCTCAACTGGATGAATGA |  |  |
| HRM 3F | GTGGGCCTTGATCTTGTTCT | 96 | HRM – Region 2 |
| HRM 3R | AAATGAGTAAGCCCAGCCTAAG |  |  |
| HRM 5F | CCGTGATCTTCTTGTTGGGATT | 112 | HRM – Region 3 |
| HRM 5R | GCAAGAAGAAGGACCCGAAC |  |  |
| S1F | TGCCACCTTCAACGACAA | 99 | HRM – Region 4 |
| S1R | ATCACGTCTGGACTGATCAAAT |  |  |
| R4F | CAAGATGATTGCCGAGCAAATG | 71 | HRM – Region 5 |
| R4R | CGCGCAAGTTCTCCCTTT |  |  |
| R6F | TTGTCGAAACCTCCCTGAC | 84 | HRM – Region 6 |
| R6R | CGGACTTGCAAAGGTGTTG |  |  |
| R3-1F | ATTCTCGTCGCGCACCT | 57 | HRM – Region 3 |
| R3-1R | CTGTCGGCCTACTTCTTCTTCT |  |  |
| R2-1F | CTAAGCGTTACCGACTAGC | 83 | HRM – Region 2 |
| R2-1R | GTCAGGTGGGCCTTGAT |  |  |

Supplementary Table 2. A list of *Phytopththora cactorum* isolates collected from strawberry in Florida, used for marker development and validation. [R indicates mefenoxam-resistant and S indicates mefenoxam-sensitive isolates for genetic regions targeted by primers set R3-1 and R2-1]

| **Isolate** | **Year of collection** | **Phenotype** | **R3-1** | **R2-1** | **DNA condition** |
| --- | --- | --- | --- | --- | --- |
| 16-88 | 2016 | Resistant | R | R | Clean |
| 16-90 | 2016 | Resistant | R | R | Clean |
| 16-91 | 2016 | Resistant | R | R | Clean |
| 16-353 | 2016 | Resistant | R | R | Clean |
| 16-363 | 2016 | Resistant | R | R | Clean |
| 16-364 | 2016 | Resistant | S | S | Clean |
| 16-365 | 2016 | Resistant | R | R | Clean |
| 18-27 | 2018 | Resistant | R | R | Clean |
| 18-39 | 2018 | Resistant | R | R | Clean |
| 18-62 | 2018 | Resistant | R | R | Clean |
| 18-627 | 2018 | Resistant | R | R | Clean |
| 18-638 | 2018 | Resistant | R | R | Clean |
| 18-639 | 2018 | Resistant | R | R | Clean |
| 18-640 | 2018 | Resistant | R | R | Clean |
| 18-641 | 2018 | Resistant | R | R | Clean |
| 18-651 | 2018 | Resistant | R | R | Clean |
| 18-656 | 2018 | Resistant | R | R | Clean |
| 18-691 | 2018 | Resistant | R | R | Clean |
| 8425 | 2019 | Resistant | R | R | Clean |
| 8477B | 2019 | Resistant | R | R | Clean |
| 8732 18 | 2019 | Resistant | R | R | Clean |
| 8732A | 2019 | Resistant | R | R | Clean |
| 8816 | 2019 | Resistant | R | R | Clean |
| 8925 | 2019 | Resistant | R | R | Clean |
| Sample 13 | 2020 | Resistant | R | R | Clean |
| Sample 19 | 2020 | Resistant | R | R | Clean |
| Sample 23 | 2020 | Resistant | R | R | Clean |
| Sample21B | 2020 | Resistant | R | R | Clean |
| 11-03 | 2011 | Sensitive | S | S | Clean |
| 13-434 | 2013 | Sensitive | S | S | Clean |
| 16-345 | 2016 | Sensitive | S | S | Clean |
| 18-31 | 2018 | Sensitive | S | S | Clean |
| 8475 | 2019 | Sensitive | S | S | Clean |
| 8729_S | 2019 | Sensitive | S | S | Clean |
| 8865_S | 2020 | Sensitive | S | S | Clean |
| 8880_S | 2020 | Sensitive | S | S | Clean |
| 8904_S | 2020 | Sensitive | S | S | Clean |
| 8912_S | 2020 | Sensitive | S | S | Clean |
| 8917_S | 2020 | Sensitive | S | S | Clean |
| 8947_S | 2020 | Sensitive | S | S | Clean |
| 8425-1 | 2020 | Resistant | R | R | Crude |
| 8425-2 | 2020 | Resistant | R | R | Crude |
| 8425-3 | 2020 | Resistant | R | R | Crude |
| 8425-4 | 2020 | Resistant | R | R | Crude |
| 8472-1 | 2020 | Resistant | R | R | Crude |
| 8472-2 | 2019 | Resistant | R | R | Crude |
| 8477-3 | 2019 | Resistant | R | R | Crude |
| 8477-4 | 2019 | Resistant | R | R | Crude |
| 8816 | 2020 | Resistant | R | R | Crude |
| 8925 | 2020 | Resistant | R | R | Crude |
| 9067-1 | 2020 | Resistant | S | S | Crude |
| 8887 | 2020 | Sensitive | S | S | Crude |
| 8889 | 2020 | Sensitive | S | S | Crude |
| 8912 | 2020 | Sensitive | S | S | Crude |
| 8920 | 2020 | Sensitive | S | S | Crude |
| 8949 | 2020 | Sensitive | S | S | Crude |

Supplementary Table 3. Function of the four genes harboring mutations associated with mefenoxam resistance in *Phytophthora cactorum,* predicted using DeepFRI, a method based on graph convolutional networks for annotating proteins and identifying functional regions in proteins (Gligorijević et al. 2021).

| **Gene ID** | **Molecular Function** | | | **Biological Process** | | | **Cellular Component** | | |
| --- | --- | --- | --- | --- | --- | --- | --- | --- | --- |
|  | **Name** | **Go Term** | **Score** | **Name** | **Go Term** | **Score** | **Name** | **Go Term** | **Score** |
| g24873 | ion binding | GO:0043167 | 1 | small molecule metabolic process | GO:0044281 | 0.95 | extracellular region | GO:0005576 | 1 |
|  | cation binding | GO:0043169 | 1 | organic substance metabolic process | GO:0071704 | 0.90 |  |  |  |
|  | metal ion binding | GO:0046872 | 1 | catabolic process | GO:0009056 | 0.78 |  |  |  |
|  | cofactor binding | GO:0048037 | 1 | organic substance catabolic process | GO:1901575 | 0.74 |  |  |  |
|  | iron-sulfur cluster binding | GO:0051536 | 0.92 | carbohydrate metabolic process | GO:0005975 | 0.74 |  |  |  |
|  | metal cluster binding | GO:0051540 | 0.92 | oxidation-reduction process | GO:0055114 | 0.72 |  |  |  |
|  | 4 iron, 4 sulfur cluster binding | GO:0051539 | 0.71 | cellular metabolic process | GO:0044237 | 0.66 |  |  |  |
|  |  |  |  | carbohydrate catabolic process | GO:0016052 | 0.65 |  |  |  |
|  |  |  |  | organic acid metabolic process | GO:0006082 | 0.60 |  |  |  |
|  |  |  |  | oxoacid metabolic process | GO:0043436 | 0.57 |  |  |  |
|  |  |  |  | organonitrogen compound metabolic process | GO:1901564 | 0.55 |  |  |  |
|  |  |  |  | drug metabolic process | GO:0017144 | 0.54 |  |  |  |
|  |  |  |  | carboxylic acid metabolic process | GO:0019752 | 0.53 |  |  |  |
| g14675 | ion binding | GO:0043167 | 1 | small molecule metabolic process | GO:0044281 | 0.93 | extracellular region | GO:0005576 | 1 |
|  | cation binding | GO:0043169 | 1 | organic substance metabolic process | GO:0071704 | 0.9 | extracellular space | GO:0005615 | 0.52 |
|  | metal ion binding | GO:0046872 | 1 | cellular metabolic process | GO:0044237 | 0.77 |  |  |  |
|  | cofactor binding | GO:0048037 | 1 | oxidation-reduction process | GO:0055114 | 0.72 |  |  |  |
|  | iron-sulfur cluster binding | GO:0051536 | 0.99 | catabolic process | GO:0009056 | 0.70 |  |  |  |
|  | metal cluster binding | GO:0051540 | 0.99 | organic substance catabolic process | GO:1901575 | 0.66 |  |  |  |
|  | 4 iron, 4 sulfur cluster binding | GO:0051539 | 0.93 | drug metabolic process | GO:0017144 | 0.64 |  |  |  |
|  | oxidoreductase activity | GO:0016491 | 0.51 | organic acid metabolic process | GO:0006082 | 0.59 |  |  |  |
|  |  |  |  | organonitrogen compound metabolic process | GO:1901564 | 0.59 |  |  |  |
|  |  |  |  | oxoacid metabolic process | GO:0043436 | 0.56 |  |  |  |
|  |  |  |  | carboxylic acid metabolic process | GO:0019752 | 0.53 |  |  |  |
|  |  |  |  | carbohydrate metabolic process | GO:0005975 | 0.52 |  |  |  |
| g11170 | ion binding | GO:0043167 | 1 | organic substance metabolic process | GO:0071704 | 0.79 | extracellular region | GO:0005576 | 1 |
|  | cation binding | GO:0043169 | 0.99 | small molecule metabolic process | GO:0044281 | 0.76 |  |  |  |
|  | metal ion binding | GO:0046872 | 0.99 | catabolic process | GO:0009056 | 0.73 |  |  |  |
|  | cofactor binding | GO:0048037 | 0.97 | oxidation-reduction process | GO:0055114 | 0.72 |  |  |  |
|  | iron-sulfur cluster binding | GO:0051536 | 0.77 | organic substance catabolic process | GO:1901575 | 0.7 |  |  |  |
|  | metal cluster binding | GO:0051540 | 0.77 | cellular metabolic process | GO:0044237 | 0.58 |  |  |  |
|  | oxidoreductase activity | GO:0016491 | 0.54 |  |  |  |  |  |  |
| g296 | ion binding | GO:0043167 | 1 | small molecule metabolic process | GO:0044281 | 0.97 | extracellular region | GO:0005576 | 1 |
|  | cation binding | GO:0043169 | 1 | organic substance metabolic process | GO:0071704 | 0.94 |  |  |  |
|  | metal ion binding | GO:0046872 | 1 | organonitrogen compound metabolic process | GO:1901564 | 0.77 |  |  |  |
|  | cofactor binding | GO:0048037 | 1 | catabolic process | GO:0009056 | 0.75 |  |  |  |
|  | iron-sulfur cluster binding | GO:0051536 | 0.96 | cellular metabolic process | GO:0044237 | 0.75 |  |  |  |
|  | metal cluster binding | GO:0051540 | 0.96 | organic substance catabolic process | GO:1901575 | 0.72 |  |  |  |
|  | 4 iron, 4 sulfur cluster binding | GO:0051539 | 0.82 | organic acid metabolic process | GO:0006082 | 0.7 |  |  |  |
|  |  |  |  | oxoacid metabolic process | GO:0043436 | 0.65 |  |  |  |
|  |  |  |  | carboxylic acid metabolic process | GO:0019752 | 0.62 |  |  |  |
|  |  |  |  | oxidation-reduction process | GO:0055114 | 0.61 |  |  |  |
|  |  |  |  | carbohydrate metabolic process | GO:0005975 | 0.6 |  |  |  |
|  |  |  |  | primary metabolic process | GO:0044238 | 0.58 |  |  |  |
|  |  |  |  | carbohydrate catabolic process | GO:0016052 | 0.57 |  |  |  |
|  |  |  |  | drug metabolic process | GO:0017144 | 0.50 |  |  |  |


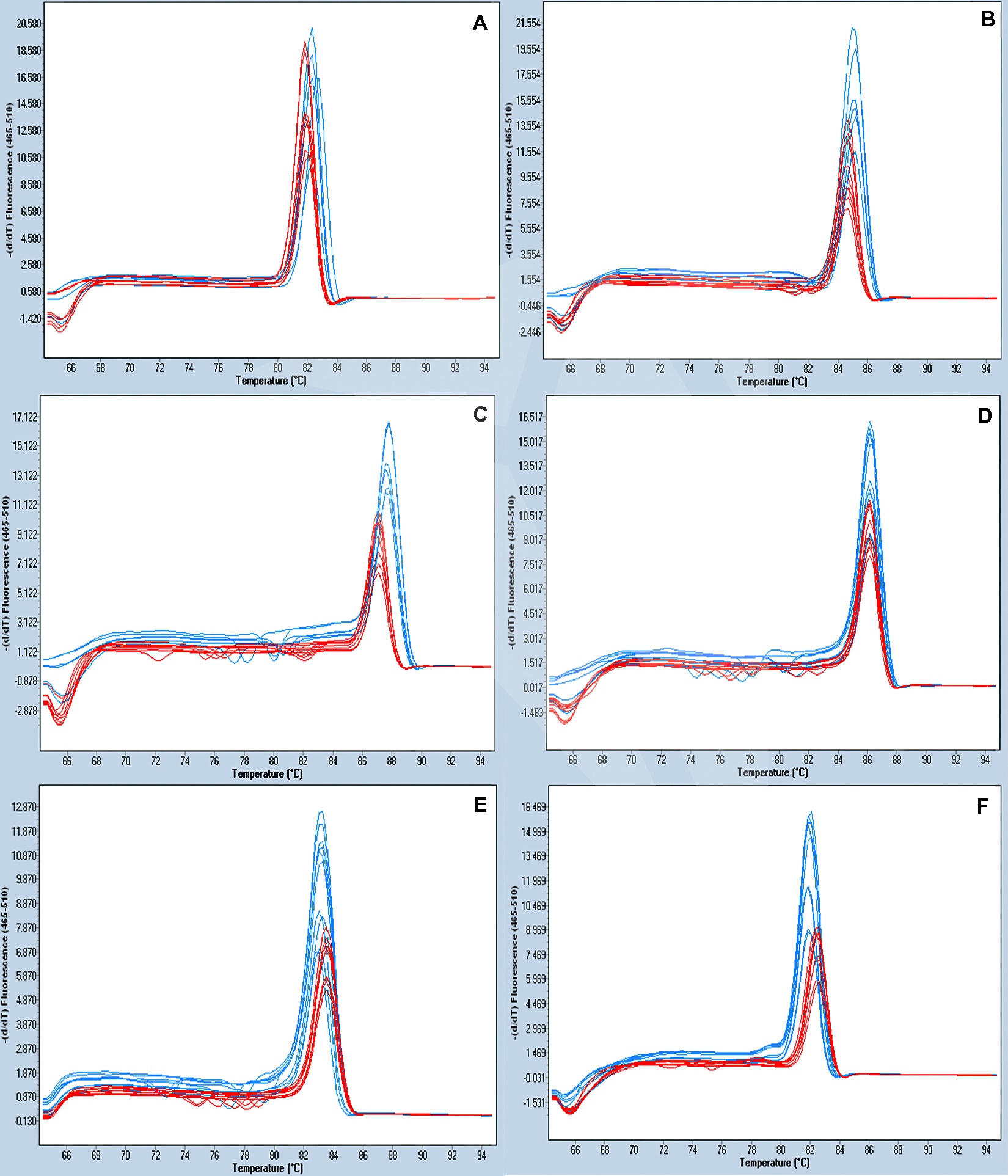


Supplementary Figure 1. High-resolution melting (HRM) analysis of regions 1 (A), 2 (B), 3 (C), 4 (D), 5 (E), and 6 (F) for the identification and differentiation of mefenoxam-sensitive (red) and -resistant (blue) isolates of *P. cactorum* of strawberry based on derivative plot analyses.


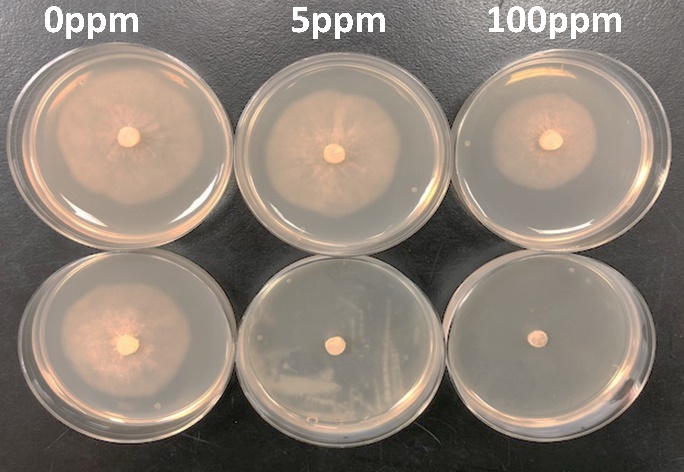


Supplementary Figure 2. Mycelial growth of a representative *Phytophthora cactorum* mefenoxam-resistant (top) and -sensitive isolate (bottom) after 4 days of plating on clarified 20% V8 medium.
